# Assessment of the Quality of Life of Vulnerable Young Males with Severe Emotional and Behaviour Difficulties in a Residential Setting

**DOI:** 10.1155/2013/357341

**Published:** 2013-12-10

**Authors:** D. Carroll, T. Duffy, C. R. Martin

**Affiliations:** ^1^Kibble Education and Care Centre, Paisley PA3 2LG, UK; ^2^University of the West of Scotland, Ayr Campus, Ayr KA8 0SX, UK; ^3^Room 2.11, Faculty of Health and Society, Buckinghamshire New University, Uxbridge Campus, 106 Oxford Road, Uxbridge, Middlesex UB8 1NA, UK; ^4^West London Mental Health NHS Trust, London UB2 4SA, UK

## Abstract

Sixty-four looked after and accommodated males aged 13–16 had an assessment of their quality of life using Paediatric Quality of Life Inventory (PedsQL) and Quality of Life in Care (QOLIC). The participants were from a Scottish residential centre for young people with severe emotional and behavioural difficulties. The total sample of 64 participants consists of two distinct groups: residential group (*n* = 33) and a secure care group (*n* = 31). Over 3 observations the aim of the study was to identify similarities and differences between the groups and to establish the sensitivity of the PedsQL and the PedsQL in care module (QOLIC) as a measurement instrument for Quality of Life (QoL) in adolescent males. Overall there was a nonsignificant increase in the quality of life of these young people at the centre as measured by PedsQL and QOLIC over 3 observations. No significant differences were detected in the quality of life scores between the two groups using the QOLIC.

## 1. Introduction

A number of studies have highlighted the poor health outcomes of looked after and accommodated children (LAAC) [1–8].

Mental health was highlighted as a particular problem with LAAC, having significantly higher incidences of mental health issues than children cared for at home [7, 9–14]. The poor physical and mental health of these young people may in the long term compound the social and economic inequality they experience by reducing their ability to achieve in education, enter the work force, and function as fully integrated members of society [15].

There is general consensus that health should be recognised in holistic terms rather than the traditional health model, with respect to people's own perceptions, attitudes, and expectations [16]. The World Health Organisation (WHO) defines quality of life as perceptions of position in life defined within the context of culture and value systems in which the person lives and with respect for their goals, expectations, standards, and concerns [17].

The aim of this study is to explore the use of Quality of Life (QoL) instruments to establish the current profile of looked after young people in Scotland. This will assist in informing the care they receive. In addition, this will enable agencies to take account of the child/adolescent perspective, serve to validate the interventions employed with these young people, and assess the outcomes to inform the development of evidence based practice.

In general, outcome measures in health have tended to focus on mortality and morbidity but Coghill et al. recommend that the focus should be on whether the person “feels” better or “is” better [18]. They conclude “it is important to acknowledge the often complex relationship between what is happening to you in your life, what you think is happening to you and how you feel about what you think is happening to you” [18, page 545].

It may be the way the individual interprets their environment that is the key factor to their QoL rating and their coping strategies in relation to that context [16]. It is accepted that the experience of being in care will have an impact on a child's physical and mental well-being. QoL can be a measure of their perception of how life is for them. There may be differences in people's views of an adolescent's quality of life. This could mean that the young person, parents, carers, social workers, researchers, and health professionals may all have a diverse view of what good health is [19–21]. It is therefore important to establish an appropriate quality of life benchmark for young people in care. There should be a minimum quality of life for young people in the care system [22].

When staff assess the young person's quality of life as being better than the young person's view, the planning and care provision to meet the young person's needs may not match. This may lead to differences in the expectations of young people and staff as the assessment may not be based on the reality of the young person's needs [20]. Within this study, some of the young people rated their quality of life higher than staff. This could be attributed to the problematic background that the young person has come from impacting on their perception of their current position. That is, their perception of their quality of life might be quite different from that of their peers and consequently may not see the need to engage in the health provisions on offer.

The effects of maltreatment on children are found in terms of the risky behaviours that they are more likely to adopt [23]. Much of the current literature highlights that these children have had negative life experiences and missed the essential health inputs from primary care services [24]. Davidson-Arad [25] focused on children who were being removed from their home and concluded that quality of life at the time was influential in 70% of the child protection officers decisions [25]. The implication is that by being received into care, quality of life would improve. However, despite being in the care system, it has been observed that young people will continue to have poor health outcomes [24].

Being in care is often believed to be the least desirable form of accommodation for children and adolescents. It is often criticised for being an institution rather than a family because of its restrictiveness and lack of permanency compared with other out of home placements [20]. The quality of life of young people in the residential care system has received little attention [20]. Assessing QoL using a reliable and valid outcome measure attributing a simple numerical value is required [26].

## 2. Background

There are a number of issues to consider when using assessment instruments that are mainly developed for adults as the health of young people is considerably different from that of adults. Young people are less able to choose and control their own environments, less likely to articulate their concerns and troubles clearly, and there is also the consideration of literacy and comprehension of questions [27].

There are two separate types of QoL instruments: generic and disease specific. Generic instruments allow comparisons to be made between different healthy populations and those with chronic health conditions. Generic instruments are used to benchmark across healthy populations and allow for comparisons across subpopulations. Disease specific instruments enhance measurement sensitivity for health domains relevant to that condition [28]. Such disease specific instruments allow clinicians to measure progress during and following treatment interventions.

QoL instruments are generally used to assess a multidimensional construct which includes domains in physical, mental, social, and psychological aspects of well-being and functioning from the young person's perspective [27]. However, there is not complete agreement on the domains of QoL. These dimensions build a profile of the young person to include moods, emotions, social support, autonomy, social acceptance and financial resources. However, it is the inter-relationship of these dimensions that affect the perceived quality of life [27].

The Paediatric Quality of Life Inventory (PedsQL) has consistently been utilised on over 25,000 children and their parents, its effectiveness reported in over 75 peer-reviewed journals publication. Using the PedsQL, young children can reliably and validly self-report their QoL [29]. There are many journal articles supporting the psychometric properties of the PedsQL: internal consistency Cronbach's alpha coefficient of >0.7 [29]; excellent factorial invariance [30]; test-retest reliability average Cronbach *α* = 0.89 [31, 32].

The assessment instrument consists of a core scale of 23 items designed to measure the physical functioning (8 items), emotional functioning (5 items), social functioning (5 items), and school functioning (5 items) aspects of QoL in healthy and ill children and adolescents. The questions are constructed in a five point format in a Likert scale from 0 (never) to 4 (almost always). There is no weighting for the items and young people are invited to focus on recalling their views over the past month. It is estimated to take five minutes to complete [33]. In a Finnish study young people reported that it was an easy instrument to complete [34].

The Quality of Life in Care (QOLIC) is a specific module of the original PedsQL. The QOLIC was developed by Upton et al. in response to the specific health needs of looked after children [24]. In their study they used this with a relatively small sample of 69 young people (aged 8–19) in public care in Wales. It is believed that there is no other instrument designed specifically to measure the quality of life of young people living in public care whose main problems have been linked to psychological health. The construct validity of this assessment instrument was analysed by Upton et al. [24] who found significant correlations between the QOLIC and the PedsQL on all subscales. In addition an acceptable internal consistency was reported with Cronbach alpha 0.87 [24].

The questionnaire consists of 19 questions on a five point Likert scale from 0 (never) to 4 (almost always). It takes less than five minutes to complete. There is no weighting for the items. Upton et al. reported that young people in public care had lower rates of quality of life than young people living at home.

## 3. Method

### 3.1. Setting

The setting for this study is the largest residential multiservice resource for LAAC in Scotland. The Centre provides specialist provision for young people with significant social and emotional needs. Local authorities typically place young people at this centre when other care establishments have not met the needs of the young person.

Two different forms of care are provided within the centre, that is, residential and secure care. Residential care is where a small percentage of children looked after by local authorities in Scotland are placed in some type of residential home or residential school. This can be provided either by the local authority or an approved independent organisation.

Secure care is a safe and controlled form of residential placement where the young person is locked up in a supportive environment in order to prevent harm to themselves or to others.

### 3.2. Participants

The centre provided the population in two groups looked after and accommodated males aged 13–16 years. The groups consisted of a residential group (*n* = 33, mean age 175 months, SD 12 months) and a secure care group (*n* = 31, mean age 178 months, SD 13 months).

### 3.3. Design

Ethical approval for the study was obtained from the University of the West of Scotland. Approval for the study was also provided by the Board of Directors for the Centre. “Opt-in” consent obtained from the young person's parent or legal guardian at the time of admission. Individual opt in consent was obtained from each young person when invited to participate in the study.

The study used a mixed-group design with the independent variables being group type (residential group/secure care group) and assessment observation point (baseline, 12 weeks and 24 weeks). The young people were assessed using the PedsQL and QOLIC quality of life assessment instrument as they entered the centre and this data collection process was repeated at 12 and 24 weeks.

### 3.4. Procedure

All the young people were administered the PedsQL and QOLIC to establish their QoL during the first week of their admission to the centre. The follow up of the QoL assessment matched the timeframe of the standard scheduled 3 monthly review process at the centre. This review at 12 and 24 weeks was in fitting with Kline's recommendations of a 3 month gap between data collection occasions [35].

The responses to both the QoL instruments are in the scale from 0 (never) to 4 (almost always). Both instruments require the child or young person to recall the past month. Therefore, the higher the score, the better the quality of life.

The study focused on 2 specific areas: to establish firstly any differences in profile of the two groups of young people in the centre and secondly identify any changes in QoL of the young people while at the centre over 12 and 24 weeks.

### 3.5. Analysis

For interpretation of the QoL scores each answered item is transformed into a rating between 0 and 100. Then the total scores are divided by the number of questions and standardised into scores out of 100.

A mixed-group 2 (between-subjects) × 3 (within-subjects) analysis of covariance (ANCOVA) procedure with age entered as a covariate was used to evaluate any statistically significant differences between the 2 groups of young people at the centre over time.

## 4. Results

Sixty-four young people completed the assessment through the 3 observations and only fully completed responses over the three observations were included in this study.

To allow for comparison between the residential and secure groups, the descriptive statistics for the PedsQL and QOLIC total scores are shown in Table 1. High scores indicate a greater quality of life.

There exists the underlying assumption that when a young person is taken into care it is to improve their quality of life [20]. The mean and standard deviation scores of the QOLIC in Table 1 show that the total scores of both groups were lowest on admission. Higher scores at the second observation and the highest scores for both groups were seen at the third observation. While this would seem reasonable it was noted that it was not a consistent trend across the individual groups over time.

The QOLIC was used to assess the QoL of the young people in residential and secure care over the three observation points. The results of the mixed-design ANCOVA for the QOLIC are shown in Table 2. No significant main effects of group type or observation time were observed, neither was there any evidence observed for an interaction between group type and observation point.

Further analysis of the results using an ANCOVA for the PedsQL (Table 3) total scores and the four subscales similarly found that no significant main effects of group type or observation time were observed, neither was there any evidence observed for an interaction between group type and observation point.

To summarise the statistical analysis, no evidence was found for statistically significant main effects of group type or observation point. There was no evidence of statistically significant interactions between group type and observation point.

## 5. Discussion 

One unique feature of this study is the assessment of the quality of life for looked after and accommodated young people using the only instrument that had been devised solely for this purpose. The scoring is a total score and there are no subscales. Upton et al. identified it as an appropriate instrument to assess looked after and accommodated young people [24].

The total scores for the PedsQL and the QOLIC showed, over time, a general trend for the increase of quality of life for both groups. This increase in quality of life scores was not statistically significant. The findings over the three observations followed the same upward trend. That is, the secure group showed a greater quality of life than the young people in residential care. The higher prevalence of quality of life in the secure group may be attributed to the stability, structures, and routine of their new secure environment. This finding using the QOLIC is consistent with the generic PedsQL instrument where the young people in the secure group perceive themselves as having a better quality of life than the young people in the residential group. This finding is not significant and this may be due to the two groups being predominately from the same population of looked after and accommodated young people. Therefore it would be worth exploring further the positive aspects for the young person perspective of being in secure care.

It is noted that despite the wide range of professional inputs the centre offers, directly and indirectly, no significant impact on their quality of life scores has been observed. This lack of significant change could relate to factors in the lives of these young people in this study. The young people have moved into a new environment away from their own community, school, support networks, friends, and family. It could therefore be expected that a decrease in their quality of life may have transpired. The quality of life of the young people has improved to a limited extent rather than deteriorated and this could be attributed to the supports provided within the centre. Assessment of quality of life over a longer period of time may demonstrate a significant positive impact once support systems become more established.

The young people are in a unique position to give their perspective on their health. A potential benefit of the PedsQL and the QOLIC instruments is that they are designed to measure the young person's perception of how s/he feels rather than having a professional assess how they are. This is in line with the Children's Act (1995) which identifies the need to provide LAAC with a voice in relation to how they perceive their difficulties [36]. Within the process of assessment, the LAAC's account is important and these assessment instruments facilitate the expression of their views. This in turn provides the opportunity to integrate the young person's perspective into care planning in order that their needs may be met.

LAAC have been a difficult group to study for many reasons. These include frequent changes in placement, social worker, and carer changes, in addition to difficulties in engaging these young people in the research process [37]. It is likely that future research will focus on several approaches to improving the outcomes of LAAC. However regardless of future approaches taken, there must be consistency in measurement of the effects of the interventions. The use of QoL instruments within this study have supported the need for consistent measures for assessing QoL for look after young people.

This study, which has identified changes of QoL over time, provides a foundation for developing a standardised framework to assess QoL of LAAC.

The use of quality of life instruments requires further investigation to understand the specific QoL domains relevant to this population to ensure the instrument captures minor changes that may take place.

The results from the analysis will provide the benchmark from which to monitor the interventions experienced by children and adolescents in public care.

## Figures and Tables

**Table 1:** Mean cores and standard deviations of the PedsQL total score and QOLIC.

QoL instrument	Group	*n*	Baseline	12 weeks	24 weeks
			mean (SD)	Mean (SD)	Mean (SD)
PedsQL total score	Residential	33	78.56 (14.31)	77.78 (12.03)	82.44 (13.59)
	secure	31	82.01 (10.71)	84.22 (8.81)	82.75 (8.49)

QOLIC	Residential	33	71.85 (15.52)	69.95 (19.20)	74.88 (21.75)
	secure	31	72.16 (15.76)	78.13 (15.24)	77.97 (11.91)

**Table 2:** ANCOVA results of QOLIC.

Source of variance	SS	df	Means square	*F *	*P* value	Partial eta squared (*n*)	Power
Within subjects							
Time	721.53	2	360.76	1.66	0.20	0.03	0.34
Time × age	632.07	2	316.04	1.45	0.24	0.02	0.31
Time × group	576.44	2	288.22	1.32	0.27	0.02	0.28
Error	26575.09	122	217.83				
Between subjects							
Intercept	2026.09	1	2026.09	4.89	0.03	0.07	0.59
Age	812.55	1	812.55	1.96	0.17	0.03	0.28
Group	612.36	1	612.36	1.48	0.23	0.02	0.22
Error	25297.75	61	414.72				

**Table 3:** ANCOVA results of PedsQL total score.

Source of variance	SS	df	Means square	*F *	*P* value	Partial eta squared (*n*)	Power
Within subjects							
Time	38.50	2	19.25	0.32	0.73	0.005	0.10
Time × age	31.06	2	15.53	0.26	0.77	0.004	0.90
Time × group	272.87	2	136.43	2.25	0.11	0.036	0.45
Error	7387.84	122	60.56				
Between subjects							
Intercept	4134.92	1	4134.92	14.55	0.00	0.19	0.96
Age	266.00	1	266.00	0.94	0.34	0.02	0.16
Group	472.18	1	472.18	1.66	0.20	0.03	0.25
Error	17337.94	61	264.23				
